# Experimental Models of Acute Lung Injury to Study Inflammation and Pathophysiology: A Narrative Review

**DOI:** 10.3390/antiox15010063

**Published:** 2026-01-02

**Authors:** Akinori Cardozo Nagato, Pedro Alves Machado-Junior, Samuel Santos Valenca, Remo Castro Russo, Frank Silva Bezerra

**Affiliations:** 1 Laboratory of Biophysics, Morphophysiology and Inflammation, Department of Biophysics and Physiology, Institute of Biological Sciences, Federal University of Juiz de Fora, Juiz de Fora 36036-900, MG, Brazil; akinori.nagato@ufjf.br; 2 Laboratory of Experimental Pathophysiology, Department of Biological Sciences and Center of Research in Biological Sciences (NUPEB), Federal University of Ouro Preto (UFOP), Ouro Preto 35402-136, MG, Brazil; pedro.junior@aluno.ufop.edu.br; 3 Institute of Biomedical Sciences, Federal University of Rio de Janeiro (UFRJ), Rio de Janeiro 21941-902, RJ, Brazil; samuelv@icb.ufrj.br; 4 Laboratory of Pulmonary Immunology and Mechanics, Department of Physiology and Biophysics, Institute of Biological Sciences, Federal University of Minas Gerais (UFMG), Belo Horizonte 31270-901, MG, Brazil

**Keywords:** acute lung injury (ALI), LPS, hyperoxia, VILI

## Abstract

Acute lung injury (ALI) is characterized by acute respiratory insufficiency, including tachypnea, cyanosis refractory to oxygen, decreased lung compliance, and diffuse alveolar infiltrates, which is a condition associated with high morbidity and mortality that usually results in the development of multiple organ dysfunction. Acute lung injury in humans is histopathologically characterized by neutrophilic alveolitis, injury of the alveolar epithelium and endothelium, hyaline membrane formation, and microvascular thrombi. Different animal models of experimental lung injury have been used to investigate mechanisms of lung injury, such as LPS-induced ALI, hyperoxia-induced ALI, and ventilator-induced lung injury (VILI). Here we will show selected preclinical mice models used as proof of concept to test new drugs in vivo with anti-inflammatory properties, discussing their particularities and clarifying the context of use.

## 1. Introduction

Acute respiratory distress syndrome (ARDS), the most devastating form of acute lung injury (ALI), is a severe clinical disorder with high mortality (30–60%) [1,2,3]. ALI is a clinical syndrome characterized by impaired gas exchange and/or lung mechanics, causing hypoxemia and increased work to breathe in patients acutely [2,3,4,5]. The risk factors for ARDS include septicemia, acid aspiration, infection, traumatic injury, fat embolism, ischemia/reperfusion, and other causes [1,2,3,5,6]. ALI is a severe clinical condition that usually results in the development of multiple organ dysfunction with high morbidity and mortality [2,3,6].

ALI/ARDS in humans is characterized by clinical features related to biological, physiological, and pathological symptoms that occur in the pulmonary tissue. Clinically, ALI is characterized by an acute onset, with diffuse alveolar injury, followed by an acute exudative phase and, consequently, tissue repair with fibrosis [7]. The pulmonary physiological changes during ALI in humans include V/Q abnormalities, severe hypoxemia that is usually responsive to positive end-expiratory pressure (PEEP) concomitant to reduced lung compliance [1,2,3,5,7], and impaired alveolar fluid clearance. The lung biological changes are marked by an increase in endothelial and epithelial barrier permeability, cytokine production, protease, and coagulation cascade activation, which are related to extravascular alveolar fluid exudation and it is associated with an impairment of the alveolar epithelium functions [1,2,3,4,5]. This is followed by pulmonary pathological changes, including increased neutrophilic alveolar infiltrates, intra-alveolar coagulation, and fibrin deposition, as well as injury of the alveolar epithelium, with denudation of the basement membrane [7].

The activation and recruitment of neutrophils are considered a central key leukocyte involved in the progression of ALI [2,3,7]. Neutrophils, innate immune cells that have anti-microbial activity, are the first leukocytes to be recruited to the site of inflammation, and the neutrophil recruitment, adhesion, migration, activation, the release of damage mediators contribute to alveolar–capillary barrier dysfunction [2,3,7]. These changes cause severe ventilation/perfusion mismatching [2,3,7,8]. Studies have demonstrated that bronchoalveolar lavage (BAL) or edema fluid aspiration from the lungs have an acute neutrophilic inflammation with fibrin-rich exudates containing a range of cytokines and chemokines [3,7]. Pro-inflammatory cytokines are produced and they include TNF-α, IL-1β, IL-6, IL-8, and IL-18, which are considered the major players in ALI and also promising biomarkers for morbidity and mortality prediction [3,5]. In order to simulate human ALI, animal models should reproduce these acute injury events and the progression of the alveolar epithelial and endothelial barrier dysfunction in the lungs, and, consequently, the acute inflammatory response in the air spaces [7]. Animal models are helpful tools and are broadly used to investigate cellular and molecular events of ALI. They are clinically relevant to humans, dissecting the cellular and molecular basis of disease in relevant and complex systems in vivo, in which ALI models are valuable as proof of concept for drug design [7].

Lipopolysaccharide (LPS), a major component in Gram-negative bacteria, has been used to induce ALI, and LPS is a potent activator of the innate immune responses via oll-like receptor 4 (TLR4) pathways [7]. LPS-induced animal models highlight ways to explore mechanisms of multiple diseases and provide helpful information on discovering novel biomarkers and pharmacological targets [9]. LPS is followed by significant increases in neutrophils in the air spaces. Thus, when delivered to animals, LPS exposure displays major features of microvascular lung injury, including leukocyte accumulation in lung tissue, pulmonary edema, profound lung inflammation, and mortality [9], which is very similar to that observed in humans.

Although oxygen (O_2_) is essential for aerobic life, it can also be an important source of cellular damage. Supra-physiological levels of O_2_ determine toxicity due to exacerbated reactive oxygen species (ROS) production, impairing the homeostatic balance of several cellular processes. Furthermore, injured cells activate inflammation cascades, amplifying tissue damage [10]. The lung is the first (but not the only) organ affected by this condition. Animal models can emulate what happens in critical care or anesthesia patients under mechanical ventilation and hyperoxia, but are also critical for exploring the effect of O_2_ on lung development and the role of hyperoxic damage and bronchopulmonary dysplasia [10].

Mechanical ventilation applies physical stresses to the tissues of the lung and thus may give rise to ventilator-induced lung injury (VILI), particularly in patients with acute respiratory distress syndrome (ARDS) [11]. The most impactful consequence of VILI is injury to the alveolar–capillary barrier, in which plasma-derived fluid and proteins leak into the airspaces and flood some alveolar regions while interfering with the pulmonary architecture and function [11]. In this narrative review, we summarize the strengths and weaknesses of different ALI models that have been used to study the mechanisms and treatment opportunities, such as LPS-induced ALI, hyperoxia-induced ALI, and VILI.

## 2. LPS (Lipopolysaccharide)-Induced Lung Injury Model

Lipopolysaccharide (LPS), a major component of cell walls from Gram-negative bacteria, is used broadly to induce ALI. The LPS is a potent activator of innate immune responses via TLR4 pathways [7,12]. LPS binds to TLR4 on the airway epithelium, leading to cytokine and chemokine production. TLR4-mediated ALI depends on TLR4/CD14/MD2 expression using the adapter proteins TIRAP and MyD88 [13]. LPS induces activation of the p38 mitogen-activated protein kinase (MAPK), and inhibition of MAPK abrogates TNF production and neutrophil recruitment into the lungs during LPS-induced lung injury in mice [13]. LPS-induced signaling in the lungs involves CD14-dependent and CD11b-dependent pathways showed to be entirely dependent on TLR4 activation, which was evidenced by the amelioration of LPS-induced acute lung injury (ALI), following early-stage TLR4 blockade [12].

LPS-induced lung injury is a very useful experimental in vivo model closely resembling ALI/ARDS in humans [9]. The intratracheal instillation of LPS is followed by an early phase characterized by a huge increase in neutrophil influx in BAL fluid after 6–8 h, and a later phase of 24–48 h after instillation with increased in BAL fluid neutrophils, monocyte, macrophage, and lymphocyte counts [7]. The neutrophil infiltration into the alveolar space is a critical event for the development of ALI, implicated in tissue damage and acute lung dysfunction [3,8]. Given the potential of targeting neutrophil migration, several small molecules have been developed to treat lung neutrophilic inflammation. Chemokines play an essential role in the migration of neutrophils, and chemokine receptors have emerged as a potential target to inhibit neutrophil accumulation [14,15]. The neutrophilic inflammation induced by LPS is abrogated in CXCR2-deficient mice, and the blockage of CXCR2 with small molecule antagonists attenuates bleomycin-induced lung injury and LPS-induced acute lung injury in mice [16,17,18]. Moreover, chemokines bind to glycosaminoglycans (GAGs) in endothelial cells, generating an immobilized chemokine gradient that directs neutrophil migration [14,15]. Some compounds that compete with chemokines for GAG binding can decrease neutrophil migration in acute lung injury models, such as bleomycin-induced lung injury [19] and LPS-induced acute lung injury in mice [20]. Targeting P-selectin and other adhesion molecules involved in neutrophil–endothelial cell interaction mediating the neutrophil migration can reduce tissue damage [3], as demonstrated in another mice model of ALI, where the PTX3 binds to P-selectin, inhibiting neutrophil influx during acid-induced acute lung injury [21]. Another potential target for the neutrophilic inflammation is the inhibition of neutrophil migration via the vascular adhesion molecule-1 blockage (VAP-1/SSAO), which attenuates acute neutrophilia and pulmonary dysfunction in LPS-induced acute lung injury and Klebsiella pneumoniae infection in mice [22]. Thus, interfering with neutrophil–endothelial cell communication modulates neutrophilic inflammation and its consequences during ALI.

LPS-induced acute lung injury can be modeled by either inhalation (intratracheal or intranasal) or systemic (intravenous or intraperitoneal) within a short period of response (usually between 6 and 48 h) [9]. Acute lung injury could be induced by an LPS single-hit (intratracheal, intranasal, intravenous and intraperitoneal), or by an LPS two-hit method. In the two-hit model, intraperitoneal injection with small doses of LPS is followed by intratracheal instillation with a median LPS dose. The LPS two-hit model seemed to be better for mimicking the pathological process of ARDS closer to the one observed in humans [9]. The susceptibility is related to genetic backgrounds and intratracheal challenge with LPS, which may vary between the production of inflammatory mediators, inflammation duration, and mice strains, such as BALB/c and C57BL/6 [23,24]. There is a difference in LPS response between different strains of the same species, i.e., BALB/c mice are very sensitive to LPS, whereas C57BL/6 mice are more resistant to LPS challenge [7].

**Figure 1:**
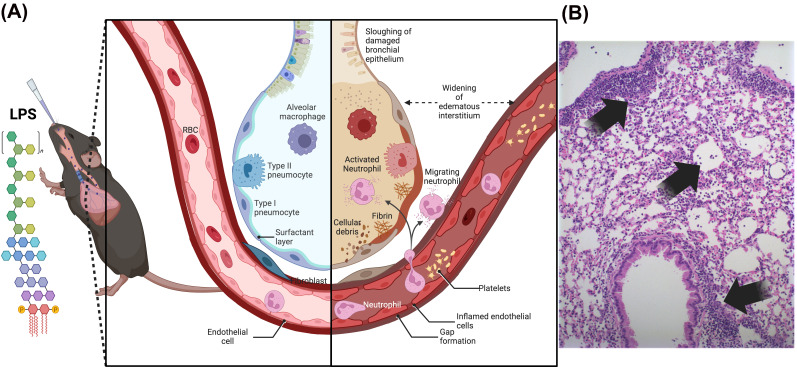
Model of LPS-induced acute lung injury by intranasal instillation in mice. (**A**) After 24 h, a neutrophilic influx into the airways occurs with exudate formation resulting from increased endothelial and epithelial barrier permeability. (**B**) Infiltration of neutrophils and a small influx of macrophages and lymphocytes found in lung tissue diffusely distributed in the airways, perivascular, and peribronchial spaces (black arrows) (photomicrography of hematoxylin- and eosin-stained lungs) and pulmonary mechanical dysfunction induced by a single intranasal instillation of LPS (25 µg/mice) in mice.

Regarding experimental models of LPS-induced acute lung injury and inflammation, there is a range of models with different LPS doses, administration routes, and number of insult repetitions, as mentioned before. The LPS-induced acute lung injury by a single dose of LPS (25 µg/mice) triggers lung inflammation between the first 6 and 24 h after intranasal instillation in C57BL/6 mice, as we previously described [20,22]. LPS-induced acute lung injury in mice is characterized by a neutrophil influx and exudation by an increase in endothelial and epithelial barrier permeability, with diffuse alveolar injury, production of cytokines, chemokines, and proteases, triggering the acute inflammatory response and coagulation in the air spaces (Figure 1A) and physiological dysfunction.

After 24 h of LPS-induced acute lung injury, there is an increased total leucocyte in BAL recovery from mice instilled with LPS, composed mostly of neutrophils and a minor influx of macrophages and lymphocytes that are found in lung tissue diffusely distributed in airways, perivascular, and peri-bronchial spaces (Figure 1B, photomicrography and arrows). This acute onset of LPS-induced acute lung injury caused by neutrophilic airway inflammation is followed by a marked reduction in lung volume depicted by functional residual capacity (FRC), decreased dynamic compliance (Cdyn), and elevation in lung resistance, as a consequence of ALI, hence mimicking pulmonary changes observed in humans. Therefore, the LPS-induced animal models reproduce acute damage to the lung epithelial and endothelial cells with the subsequent acute inflammatory response in the air spaces and dysfunction [3], thus being considered a valuable tool to study new anti-inflammatory drugs.

## 3. In Vivo Animal Model of Hyperoxic-Induced Oxidative Stress

The late 18th and early 19th centuries were marked by the discovery of oxygen (O_2_), the beginning of its use in experimental and clinical models, and the first evidence of its toxic effects, marked especially with lower airway inflammation [25]. The discovery of O_2_ occurred in 1771 with studies on combustion. Carl Sheele observed that the presence of a previously unknown gas in the atmosphere was necessary for combustion to occur—this theory became known as phlogistic theory [26]. Afterward, in 1774, Joseph Priestley found that the gas thought to be generated during combustion was consumed. Contrary to the previous idea, Priestley denominated the O_2_ gas as “dephlogisticated” and suggested that this gas could potentially promote adverse effects on the organism. The definitive nominal description of oxygen (O_2_) and its chemical properties, such as its mass, was only later defined by Antoine Laurent Lavoisier [26].

The discovery of oxygen not only accompanied the scientific revolution and the beginning of modern chemistry, it also revolutionized the therapy for patients with hypoxemia, since once isolated and stored in high concentration, the O_2_ could also be offered in inspired oxygen fractions larger than those of the atmosphere and for a prolonged period. However, along with these advances, studies sought to investigate the adverse effects of oxygen due to its toxicity [26,27].

Paul Bert (1878) was a pioneer in studies of oxygen toxicity when he demonstrated that changes in the central nervous system in humans, such as convulsions (known as the “Bert effect”), resulted from exposure to atmospheric air at 15–20 ATA (absolute atmosphere). From an experimental point of view, it was Lorrain Smith (1899) who observed the first evidence of the onset of fatal pneumonia in rats exposed for 4 days to 73% O_2_ at 1 ATA (this became known as the “Smith effect”) [28].

Numerous experiments have been performed throughout the 20th and 21st centuries with different animal species (rabbits, cats, dogs, primates, rats, and mice), varying O_2_ exposure times, and oxygen concentrations, which have corroborated the first hypotheses about its toxicity and lethality [10]. However, the mechanisms of O_2_-induced acute lung injury are still unclear. Evidence that even genetic backgrounds (at least of mice) can modify the response to hyperoxia further broadened the spectrum of investigations and reinforced the implementation of different experimental models [29,30].

Old studies pointed out that a fraction of 0.7 inspired O_2_ (FIO_2_) seemed to be the limit between a protective effect or beyond, in which toxic effects would be clinically relevant [31]. However, recent studies have contradicted these observations, showing that inspired oxygen fractions at low or moderate concentrations are also potentially deleterious, especially to the lungs [32]. Oxygen therapy is among the most common therapeutic interventions worldwide [33], and it aims to maintain the ideal PaO_2_ of the individual to reverse the installation of hypoxemia, as well as to maintain physiological functions dependent on O_2_, such as cellular respiration, and generation (at normal levels) of reactive oxygen species (ROS) in order to regulate cell growth, proliferation, differentiation, survival, and even apoptosis. The estimated PaO_2_ value for each patient results from the equation PaO_2_ = 109 − 0.43 X age [34].

Insufficient O_2_ supply accentuates hypoxemia and, on the other hand (paradoxically), supply beyond ideal levels, and especially for an extended period, leads to hyperoxemia and hyperoxia, which predisposes the organism to the consequences of its toxicity. The installation of acute respiratory failure type I and II, cardiovascular diseases, coma, shock, and chronic diseases such as cystic fibrosis and COPD, for example, are conditions that predispose the body to greater vulnerability and undesirable effects from hyperoxia, since in these conditions they become refractory in the attempts to reverse hypoxemia, and the supra-physiological supply of O_2_ is often necessary to minimize the imminent risk, including death [35]. Therefore, adjusting the optimal FiO_2_ levels makes it difficult, leading to iatrogenic [10].

The O_2_ administration device directly influences the FiO_2_ and the final O_2_ concentration offered to the cells. Open systems of the O_2_ offer are more variable, such as nasal catheters (FiO_2_ = 24 and 40%), nasal masks (FiO_2_ of up to 60%), newborn hoods (FiO_2_ of 100%), and an oxygen tent (FiO_2_ of up to 60%) [36,37,38,39], while closed systems are precise (as in artificial mechanical ventilation and hyperbaric O_2_ chambers), and are potentially more harmful [31]. Experimental hyperoxia models have studied the effects of O_2_ by subjecting organisms to elevated O_2_ concentrations, for varying amounts of time, through different oxygen delivery devices and under different genetic and inflammatory disease backgrounds. In these models, the lungs have been the primary organs of interest since they are primary interfaces for O_2_ and are certainly exposed to higher O_2_ concentrations than any other organ of the organism that has the airways as the direct route of O_2_ introduction [7,10,32]. For this reason, the information in this chapter was directed primarily toward the experimental hyperoxia model, which utilizes the lungs as the object of investigation.

The toxic potential of oxygen lies in the fact that it destabilizes the cellular oxide-reduction balance towards the excessive formation of reactive oxygen (ROS) and nitrogen (NOS) species. Under increased O_2_ bioavailability, flavins, quinones, and NADPH oxidases reduce a greater amount of O_2_ to superoxide anion (O_2_-), which leads to greater dismutation to H_2_O_2_ by the enzyme superoxide dismutase (SOD) and a greater amount of hydroxyl radical (HO-) is formed. The excess of ROS and NOS characterizes oxidative stress. However, due to the short half-life [33], experimentally, this imbalance is estimated by oscillating the activity of non-enzymatic antioxidant components (glutathione system) [40] of the antioxidant enzymes (CAT, SOD, GPx) [41], or the increased presence of transcription factors for these enzymes [42,43], such as Nrf2, or their activation pathways, such as MAPK/ERK [44]. The presence of O_2_ also accentuates the ONOO-formation mediated by the enzyme nitric oxide synthase, which accelerates the conversion of NO and O_2_ in the presence of L-NAME [45].

Sustained, hyperoxia-induced oxidative stress triggers pulmonary inflammation mediated by NF-kB [46], IL-8, and TNF-alpha [47], as well as the high-mobility group box-1 (HMGB1) [48], and promotes oxidative damage, with a substantial presence of lipid peroxidation sub-products such as MDA [49], carbonyl groups [50], and DNA base adducts 8-Oxo-2′-deoxyguanosine (8-oxo-dG) [51], which induce activation of the apoptotic cascade, with a marked presence of caspases and c-Jun N-terminal kinase (JNK) [52,53] and tissue necrosis [54]; and there is evidence that it is superimposed on already installed inflammation, and hyperoxia-induced oxidative stress polarizes the immune cell phenotype [55].

The implementation of an in vivo animal model of hyperoxia-induced oxidative stress can provide essential insights into the role of oxide-reduction stability on tissue proliferation and growth, as well as the pathways by which sustained oxidative stress leads to or modifies the course of inflammation, induces apoptosis, and oxidative damage which is not only pulmonary, but also in other organs. This complex scenario has directed the implementation of experimental hyperoxia models in three crucial and different didactic directions. First, models that are implemented aim to study the effects of stress, oxidative damage, and inflammation induced by hyperoxia, varying time and O_2_ concentration superimposed on different species of animals. These models seek to make indirect inferences with human models where oxygen therapy is used without a prior inflammatory background. Second, there are experimental models where hyperoxia serves the researcher with a means to induce stress and oxidative damage superimposed on other pre-existing inflammatory insults, e.g., when hyperoxia is superimposed on experimental models of asthma, emphysema, and pulmonary fibrosis. This line of study seeks to mimic clinical situations where oxygen is administered and superimposed on a usually pre-existing inflammatory condition. Third and last, models of hyperoxia-induced oxidative stress and damage have served as an oxidative background for testing biomodulators of the oxide-reduction balance. In this sense, the implantation of the experimental hyperoxia model is particularly interesting because oxygen, being a fat-soluble biomolecule, is able to permeate through all organic tissues (it is not recognized as a foreign molecule to the organism), and it can induce stress and oxidative damage without triggering an immediate immune response. Future directions, perhaps, with the use of methods that maintain oxidative stress sustained for an extended period and in low concentration, may help clarify how organisms adapted evolutionarily to the variations in oxygen concentration in the atmosphere.

In hyperoxia-induced lung injury models, excessive oxygen exposure triggers an imbalance between oxidant generation and antioxidant defenses, leading to pronounced oxidative stress and inflammation. Elevated oxygen tension enhances the mitochondrial production of reactive oxygen species (ROS), including superoxide anions, hydrogen peroxide, and hydroxyl radicals, which damage cellular macromolecules such as lipids, proteins, and DNA. This oxidative insult results in increased lipid peroxidation, accumulation of protein carbonyls, and activation of redox-sensitive transcription factors like NF-κB and Nrf2 [56].

**Figure 2:**
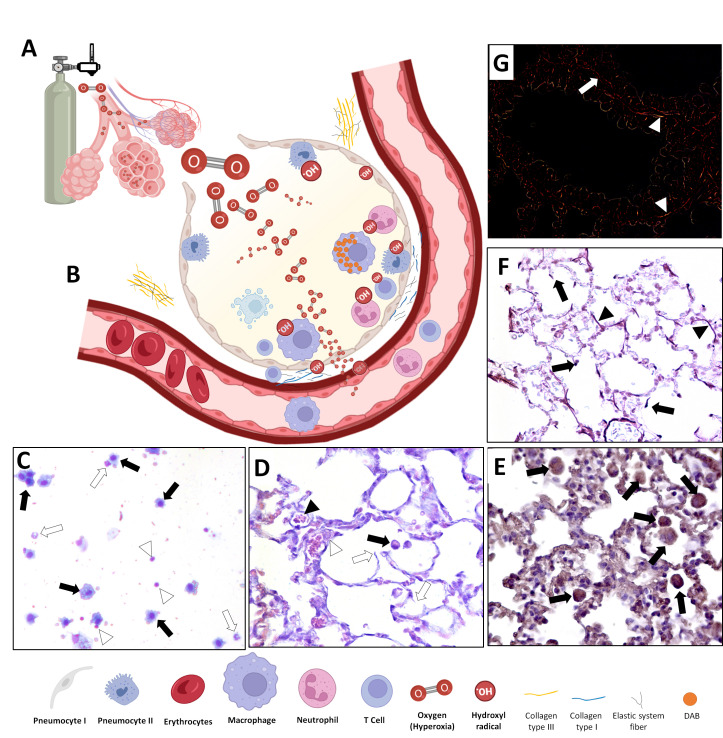
The initial phase of oxygen exposure, induction of oxidative stress, induced inflammation, and hyperoxia-induced lung damage. (**A**) An example of oxygen delivery using a cylinder and Thorpe and Bourdon tubes and exposure of the airway and alveoli to hyperoxia. (**B**) Oxygen reaches type I and II pneumocytes, and alveolar macrophages, and induces oxidative stress with final hydroxyl radial formation; it recruits lymphocytes and neutrophils to the alveoli, activates cellular responses, as in macrophages, and results in cellular (e.g., in macrophages) and tissue (pneumocytes) damage. Hematosis is intensified, and a supra-physiological systemic oxygen concentration is sustained while exposure to hyperoxia remains. Twenty-four hours of hyperoxia at 100% induces macrophage, neutrophil, and lymphocyte recruitment to the airways and lungs, activates macrophages, modifies the extracellular matrix, and promotes lung damage. (**C**) Macrophages (black arrows); neutrophils (white arrows); lymphocytes (arrowheads) collected in bronchoalveolar lavage (stained in rapid panoptic–cytospin method). (**D**) Macrophages (black arrows); alveolar septa lesions (white arrows); vessels (black arrowheads); monocyte (white arrowheads); in lung tissue stained in hematoxylin and eosin. (**E**) Alveolar macrophages expressing t-Bet transcription factor after immunohistochemistry assay (black arrows); (**F**) intact elastic fibers (black arrows); (**G**)-(Picrosirius red)-Type III collagen-yellowish appearance (white arrowhead). Type I collagen-intense reddish appearance (white arrow).

Consequently, there is a marked upregulation of pro-inflammatory cytokines (TNF-α, IL-1β, IL-6) and chemokines (CCL2), promoting leukocyte recruitment—particularly neutrophils—into the alveolar space (Figure 2). These infiltrating cells amplify tissue injury through the release of proteases and additional ROS, exacerbating epithelial and endothelial damage. Alveolar–capillary barrier disruption follows, leading to pulmonary edema, thickening of alveolar septa, and impaired gas exchange.

At the same time, endogenous antioxidant defenses, such as glutathione (GSH), superoxide dismutase (SOD), and catalase, become depleted, compromising the cellular capacity to neutralize ROS. Histologically, these lungs exhibit alveolar collapse, inflammatory infiltrates, and structural disorganization of the parenchyma. Overall, hyperoxia exposure induces a self-perpetuating cycle of oxidative damage and inflammation, culminating in tissue remodeling and respiratory dysfunction. Interventions aimed at enhancing antioxidant capacity or modulating redox signaling have shown promise in mitigating these deleterious effects [56].

## 4. Experimental Models of Ventilator-Induced Lung Injury Model (VILI)

Mechanical ventilation (MV) has been a life-saving therapy used in critical care patients for several decades [57,58]. MV improves gas exchange as well as preserves our life when the ventilatory muscles are unable to maintain lung ventilation in the conditions of acute or chronic respiratory failure [59]. Each year, thousands of people are subjected to mechanical ventilation, and this type of life support is essential for critically ill elderly patients [60]. However, MV is an anti-physiology tool that promotes overdistension on lung units by opening and collapsing the atelectatic areas of the lung, leading to inflammatory cell recruitment as well as redox imbalance that must result in ventilator-induced lung injury (VILI) [56,61]. Ashbaugh et al. described the adult (acute) respiratory syndrome (ARDS) in 12 patients with tachypnea, hypoxemia, and, consequently, loss of compliance with atelectasis, which was more helpful with the positive end-expiratory pressure (PEEP) concerning atelectasis and hypoxemia [62]. However, it was only in 1974 that Webb and Tierney performed an experimental study with rats, demonstrating that in 35 min under MV and with a peak airway pressure at 45 cm H_2_O, they can develop profuse pulmonary edema and alveolar flooding [63]. However, in 1964, Greenfield et al. evaluated the effects of MV during 2 h at 26–32 cm H_2_O of peak inspiratory pressure in dogs. They found zones of atelectasis, thus suggesting altered surfactant properties [64]. Subsequently, the replication of these observations caused by high airway pressure in larger animals (i.e., rabbits, sheep, dogs, and lambs) required a long period of MV [65,66,67,68,69]. Another critical variable used to cause the development of VILI is volume (tidal volume = Vt) [70]. High Vt promotes overstretching of the lungs as well as an increase in the mortality of critical care patients under MV (~Vt = 12 mL/kg versus ~Vt = 6 mL/kg) [71]. From experimental models in VILI, the use of ~Vt = 12–20 mL/kg induces stretching within mouse lungs [70]. These associations between high airway pressure and/or high tidal volume trigger a pulmonary inflammatory response with the recruitment of leukocytes as well as the release of inflammatory mediators with the production of local and systemic reactive species of oxygen/nitrogen, which is called biotrauma [7,56,72].

The cellular and biochemical mechanism associated with the development and progression of VILI is mechanotransduction, such as cell deformation [61]. The primary regulatory cells in VILI are alveolar macrophages, alveolar epithelial cells (AECs), and neutrophils [56,61]. Alveolar macrophage is responsible for mechanical stress and the production of different interleukins such as IL-8, Metalloproteinase 9 (MMP9), and activation of NFkB caused by cyclic pressure stretching [73]. Regarding alveolar epithelial cells, which are associated with the production of TNF caused by MV, mechanotransduction promotes cellular membrane damage via Ca^2+^ in AECs [74,75]. For example, type I epithelial cells, when kept under cyclic stress caused by MV, increased the generation of reactive species in oxygen/nitrogen, such as superoxide and nitric oxidase [76]. Therefore, the generation of reactive species is associated with monolayer permeability via NF-κB activation and ERK phosphorylation [72].

Specific inflammatory mediators are associated with cyclic stress caused by MV. High-mobility group box protein 1 (HMGB-1), which is a non-histone nuclear protein, is associated with the triggering of several pro-inflammatory cytokines such as TNF-alpha and IL-8, and monocyte chemotactic protein 1 (MCP-1) is expressed by AECs and alveolar macrophages [56,77]. Consequently, when MV contributes to VILI, it promotes the recruitment of neutrophils from the bloodstream into the airways. The activation of neutrophils is initiated by TNF-alpha and IL-1 beta, which contribute to the development and progression of VILI, specifically in mice [56]. Neutrophils produce granules that may contain myeloperoxidase (MPO), NADPH oxidase, MMP9, and neutrophil elastase (NE). Therefore, these enzymes are essential in producing ROS and activating MMPs, leading to the degradation of pulmonary parenchyma caused by MV [56,72].

**Figure 3:**
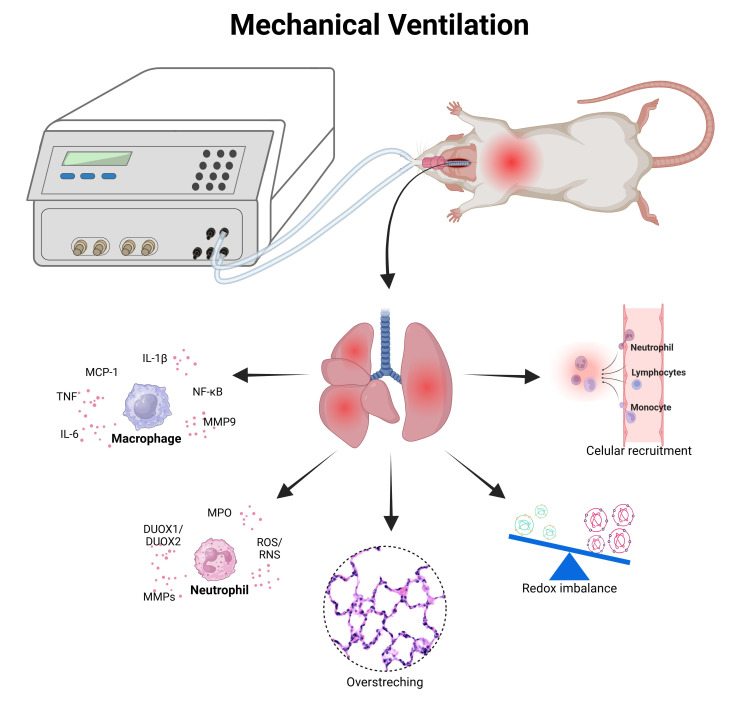
Created by Biorender. Cellular and molecular mechanisms involved with VILI. Abbreviations: interleukin (1β); IL-6; monocyte chemotactic protein 1 (MCP-1); tumor-necrosis factor (TNF); nuclear factor kappa B (NF-kB); matrix metalloproteinase 9 (MMP9); myeloperoxidase (MPO); duol oxidase 1 (DUOX1) and 2 (DUOX2); metalloproteinases (MMPs); reactive oxygen species (ROS); reactive nitrogen species (RNS).

The role of neutrophils is essential for the development and/or progression of ALI/ARDS [78]. These inflammatory cells participate directly in this process since neutrophils produce Duol oxidases (DUOX1 and DUOX2), which are enzyme members of the membrane-bound nicotinamide adenine dinucleotide phosphate oxidase family, which are found on the epithelial surface of the lung and perform the integration and signaling during different inflammatory processes [79] (Figure 3).

Regarding experimental models of VILI, there are basically two models: (a) experimental models of VILI with direct insult from lungs (LPS, oleic acid, hydrochloric acid (HCL), α-naphthylthiourea (ANTU), warm saline, meconium solution, and dioctyl sodium sulfosuccinate detergent (aerosol)) (see Table 1); (b) experimental models of VILI in healthy lungs, i.e., critical physical forces causing VILI such as stress (force per unit of area) and strain (force along the longitudinal axis). These two mechanisms directly damage the alveolar–capillary barrier and ECM or promote a mechanical stimulus into intracellular biochemical and molecular signals [72]. Indirect acute lung injury (ALI), frequently associated with extrapulmonary sepsis, is characterized by a systemic insult that primarily targets the pulmonary vascular endothelium. This process differs from direct ALI caused by aspiration, in which the initial damage occurs in the alveolar epithelium. In both cases, the inflammatory response culminates in disruption of the alveolar–capillary barrier, leading to pulmonary edema and respiratory failure [80].

Regarding the experimental models of VILI with direct insult from lungs, there are several limitations, such as the use of high-positive end-expiratory pressure, the use of oxygen in high concentration (hyperoxia), as well as the use of large tidal volume in small or large animals under MV. However, our research group aimed to evaluate and establish experimental models of VILI using healthy lungs, which means performing only the isolated effects of MV without variables (high PEEP or hyperoxia) with a focus on oxidative stress and inflammation in the lungs of healthy small animals [81]. Basically, we are working with rats and mice and since 2018, we have demonstrated the association between redox imbalance and pulmonary inflammation (acute lung injury) with only one hour of MV (Figure 3) using different models of VILI without lung injury [82,83,84,85,86,87].

**Table 1:** Chronological order of the leading experimental models of ventilator-induced injury with previous lung injury.

Animal Models	Type of Injury	Dose	Route
Guinea-pigs	Physiological saline	35 mL/kg [88]	Intratracheal
Mongrel dogs	Oleic acid	0.06 mL/kg [89]	Intravenous
Mongrel dogs	Detergent dioctyl sodium sulfosuccinate	1.5 mg/kg [90]	Aerosolization
New Zealand white rabbits	Warm normal saline	30 mL/kg [91]	Intratracheal
Mongrel dogs	Hydrochloric acid	0.5 mL/kg [92]	Intratracheal
New Zealand white rabbits	Hydrochloric acid	2.25 mL/kg [93]	Intratracheal
New Zealand white rabbits	Detergent dioctyl sodium sulfosuccinate	3.8 mg/kg [94]	Intratracheal
New Zealand white rabbits	Warm normal saline	30 mL/kg [95]	Intratracheal
Wistar rats	α-naphthylthiourea	1.3 mg/kg [96]	Intravenous
Yorkshire pigs	Warm saline solution	50 mL/kg [97]	Intratracheal
Wistar rats	Paraquat	15 mg/kg [98]	Intraperitoneally
Yorkshire pigs	Warmed saline 0.9%	30 mL/kg [99]	Intratracheal
Wistar rats	Lipopolysaccharide	200 μL [100]	Intratracheal

In models of ventilator-induced lung injury (VILI), oxidative stress and inflammation play central roles in the progression of tissue damage [101]). Mechanical ventilation with high tidal volumes or pressures promotes excessive production of reactive oxygen (ROS) and nitrogen species (RNS), activating pro-inflammatory pathways such as NF-κB and increasing iNOS expression. These events lead to endothelial dysfunction, enhanced vascular permeability, and pulmonary edema formation. In parallel, antioxidant defenses become compromised, evidenced by reduced levels of reduced glutathione (GSH), a lower GSH/GSSG ratio, and decreased activity of enzymes such as superoxide dismutase (SOD) and catalase [56]).

**Figure 4:**
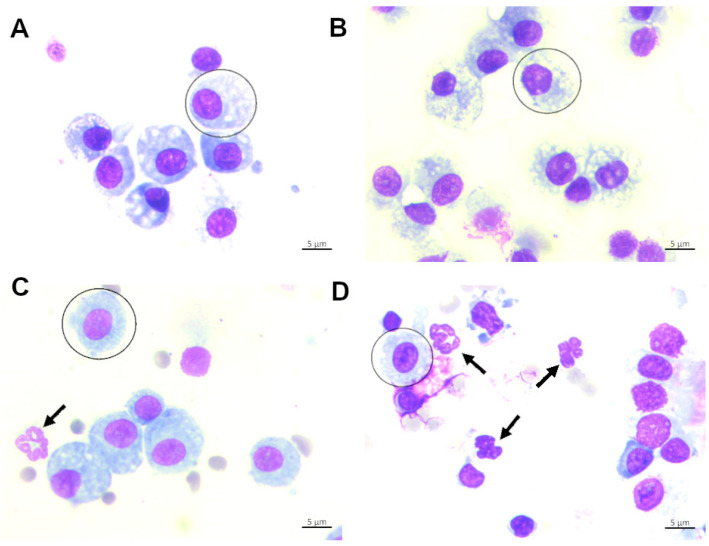
Photomicrographs of the influx in inflammatory cells from the bronchoalveolar lavage fluid of animals under mechanical ventilation. (**A**) Control group; (**B**) animals under mechanical ventilation with a tidal volume of 4 mL/kg; (**C**) animals under mechanical ventilation with a tidal volume of 8 mL/kg; (**D**) animals under mechanical ventilation with a tidal volume of 12 mL/kg. The circle indicates macrophage, and the arrows show the neutrophil.

This redox imbalance results in lipid peroxidation, protein carbonylation, and apoptosis of alveolar cells, ultimately causing disorganization of pulmonary architecture. Moreover, oxidative stress contributes to the activation of inflammatory pathways and recruitment of immune cells, such as neutrophils and macrophages, amplifying inflammation and perpetuating the cycle of tissue injury (Figure 4). Therefore, structural remodeling of the parenchyma occurs, accompanied by loss of alveolar–capillary barrier integrity and impairment of respiratory function. Experimental studies demonstrate that antioxidant interventions, such as N-acetylcysteine or SOD mimetics, can mitigate these responses by restoring redox balance, reducing inflammation, and preserving alveolar integrity. Therefore, in VILI models, a significant increase in oxidative stress markers, reduction in endogenous antioxidant defenses, elevation in inflammatory cytokines, and histological evidence of structural damage are expected—effects that can be partially reversed by antioxidant strategies [56].

There are different experimental models of VILI with previous or no lung injuries. However, some variables, such as PEEP, high oxygen, high respiratory rate, and high airway pressure, must be avoided during MV. These variables cause bias in results and change the future of different investigations into VILI. Another critical point here is the pre-treatment or treatment associated with experimental models of VILI. We believe that bioactive molecules such as hesperidin, lycopene, n-acetylcysteine, and exogenous surfactants (especially combined with nano molecules) are the strongest candidates to use in clinical trials in the near future.

## 5. Conclusions

In summary, our narrative review highlights the importance of different experimental models of acute lung injury, as well as the mechanisms associated with good protocol practices and the use of animals in preclinical studies. Understanding and standardizing animal use as well as its procedures in basic research is fundamental for advancing science with accuracy, transparency, and efficacy.

## Data Availability

No new data were created or analyzed in this study. Data sharing is not applicable to this article.

## Abbreviations

The following abbreviations are used in this manuscript:

ALI	Acute lung injury
AECs	Alveolar epithelial cells
ARDS	Acute respiratory distress syndrome
BAL	Bronchoalveolar lavage
CAT	Catalase
GAGs	Glycosaminoglycans
GPx	Glutathione peroxidase
HMGB1	High-mobility group box-1
JNK	c-Jun N-terminal kinase
LPS	Lipopolysaccharide
MAPK	Mitogen-activated protein kinase
MDA	Malondialdehyde
MV	Mechanical ventilation
NOS	Nitrogen species
PEEP	Positive end-expiratory pressure
RBCs	Red blood cells
ROS	Reactive oxygen species
SOD	Superoxide dismutase
VILI	Ventilator-induced lung injury
